# Prediction model based on preoperative CT findings for carotid artery invasion in patients with head and neck masses

**DOI:** 10.3389/fonc.2022.987031

**Published:** 2022-10-06

**Authors:** Yanfeng Zhao, Dan Bao, Xiaoyi Wang, Meng Lin, Lin Li, Zheng Zhu, Xinming Zhao, Dehong Luo

**Affiliations:** ^1^ Department of Radiology, National Cancer Center/National Clinical Research Center for Cancer/Cancer Hospital, Chinese Academy of Medical Sciences and Peking Union Medical College, Beijing, China; ^2^ Department of Radiology, National Cancer Center/National Clinical Research Center for Cancer/Cancer Hospital and Shenzhen Hospital, Chinese Academy of Medical Sciences and Peking Union Medical College, Shenzhen, China

**Keywords:** carotid artery, computed tomography, head and neck tumors, retrospective study, risk factors, regression analysis

## Abstract

**Objectives:**

To investigate the performance of a model in predicting carotid artery (CA) invasion in patients with head and neck masses using computed tomography (CT).

**Methods:**

This retrospective study included patients with head and neck masses who underwent CT and surgery between January 2013 and July 2021. Patient characteristics and ten CT features were assessed by two radiologists. The patients were randomly allocated to a training cohort (n=106) and a validation cohort (n=109). Independent risk factors for CA invasion were assessed by univariate and multivariate logistic regression analyses. The predictive model was established as a nomogram using the training cohort. In addition, the calibration, discrimination, reclassification, and clinical application of the model were assessed in the validation cohort.

**Results:**

A total of 215 patients were evaluated, including 54 patients with CA invasion. Vascular wall deformation (odds ratio [OR], 7.17; p=0.02) and the extent of encasement to the CA (OR, 1.02; p<0.001) were independent predictors of CA invasion in the multivariable analysis in the training cohort. The performance of the model was similar between the training and validation cohort, with an area under the receiver operating characteristic curve of 0.93 (95% confidence intervals [CI], 0.88-0.98) and 0.88 (95% CI, 0.80-0.96) (p=0.07), respectively. The calibration curve showed a good agreement between the predicted and actual probabilities.

**Conclusion:**

A predictive model for carotid artery invasion can be defined based on features that come from patient characteristics and CT data to help in improve surgical planning and invasion evaluation.

## Introduction

The surgical treatment of head and neck masses involving the carotid artery (CA) is challenging (1). In cases of invasion of the common or internal CA, treatment strategies include arterial resection with or without graft replacement, excising carotid body tumors for cure or palliation, and nonsurgical approaches, such as chemotherapy and radiotherapy (2). The complete surgical removal of head and neck tumors cannot be achieved without arterial resection. However, the poor prognosis of these patients, the risk of surgical complications, and the high incidence of distant metastases are reasons to refrain from surgery (1, 3–5). Despite controversies regarding the best management of these situations, the aggressive treatment of tumors involving the CA improves locoregional control (4, 6).

The invasion of the common or internal CA can be visualized using imaging techniques, including ultrasound, magnetic resonance imaging (MRI), and computed tomography (CT). Lodder et al. demonstrated that the preoperative assessment of the encasement of the internal CA using MRI or CT had a false-negative rate of 1.5% (7); however, intra-observer variability was high (7–10). Most studies on the preoperative imaging of CA involvement evaluated a small number of cases (less than 100) and a single abnormal imaging sign (9, 11, 12).

Li et al. (13) preoperatively evaluated peripancreatic arterial and venous invasion based on multi-detector row CT signs in 54 patients eligible for the surgical treatment of ductal pancreatic carcinoma and found that tumor invasion was greater than 50% in 97% (28/29) of the affected arteries. The positive predictive value (PPV), sensitivity, and specificity of predicting hepatic artery involvement in perihilar cholangiocarcinoma (14) on CT considering four criteria (arterial encasement >180°, narrowing, irregularity, and occlusion) was 53%, 40%, and 75%, respectively. Encasement of ≤270°, tumor contact length of ≤26 mm, tortuosity, occlusion, and stenosis predicted artery invasion (14–16). This study compared the CT signs of CA invasion with signs of tumor invasion of other arteries. Tumor invasion may depend on vascular wall elasticity, structure, and thickness.

Predictive models are widely used in differential diagnosis and prediction of tumor staging, treatment efficacy, and patient prognosis (17–20). However, to our knowledge, there is no consensus on the ability of these models to predict CA invasion.

This study evaluated the ability of a model to predict CA invasion by head and neck masses using CT and assessed the clinical utility of a nomogram based on this model.

## Materials and methods

### Patients

This retrospective study was approved by the Institutional Review Board of our hospital, and the need for informed consent was waived because of the retrospective nature of the study. Consecutive patients who underwent surgical treatment for head and neck tumors between January 2013 and July 2021 were evaluated retrospectively. The inclusion criteria were: (1) benign or malignant tumors involving the CA, (2) tumors in arteries other than CAs, (3) surgery within 14 days after CT examination, (4) definite intraoperative or pathological diagnosis of CA involvement, (4) complete preoperative CT imaging data; and (5) age >18 years (patients younger than 18 years were included under guardians’ consent). In total, 215 patients were included in the study and were randomly allocated to a training set (106 patients) or a validation set (109 patients) at a ratio of approximately 1:1.

### CT examination

All patients underwent CT examination using a 64-slice spiral CT scanner (Lightspeed; VCT or Discovery HD750; GE Healthcare, US) in the supine position. Scans were performed from the skull base to the superior border of the manubrium. After plain CT, the patients received a contrast agent (1.2 mL/kg; Omnipaque 350 mg I/mL; GE Healthcare, US) at a flow rate of 3.5 mL/s, followed by 40 mL of saline solution through the elbow vein using a power injector (Medrad Stellant, Indianola, PA) at a flow rate of 3.0 mL/s. The arterial and venous phases were 15 and 45 s after contrast agent injection. Images were obtained using the following parameters: tube voltage, 120 kV; tube current, 340 mA; slice interval, 5 mm; slice thickness, 5 mm; reconstruction slice, 1.25 mm, interval, 0.8 mm; rotation time, 0.6-0.7 s; helical pitch, 0.984, matrix, 512 × 512; field of view, 24 cm. Images were reconstructed using a standard algorithm. Images were interpreted using multiple planar reconstruction and maximum intensity projection.

### Imaging analysis

Junior and senior head and neck radiologists blinded to the histopathological findings of CA invasion retrospectively and independently reviewed CT images.

**Figure 1:**
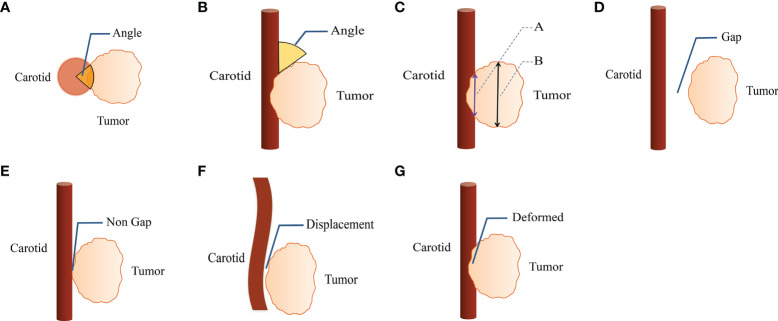
CT imaging parameters are included in qualitative analysis. **(A)** The degree of encasement of vascular structure (axial surrounding angle). **(B)** The angle between the mass and the vascular structure is longitudinal to the vascular structure (longitudinal axis angle). **(C)** Line A is the length of the interface between tumor and carotid artery along the long axis of vascular structure, line B is the length of the tumor along the long axis of vascular structure. **(D, E)** The perivascular fatty gap between the carotid artery and the tumor. **(F)** Vascular displacement. **(G)** Vascular deformation.

The following imaging parameters were evaluated for qualitative analysis: (i) vascular encasement (axial angle) (0-360°); (ii) angle between the tumor mass and the CA longitudinal to the vessel (longitudinal axis angle) (0-180°); (iii) length of the interface between the tumor and the CA longitudinally; (iv) length of the tumor along the vessel; (v) length of the interface between the tumor and CA longitudinally divided by tumor length along the vessel (iii/iv). (**Figure 1**) The presence of perivascular adipose tissue, vascular abutment by tumors (resulting in changes in vascular position), and vascular deformation by tumors (resulting in morphological changes) was evaluated. The sites of head and neck masses and side (left/right) of the affected CA were also analyzed. Interobserver agreement of imaging findings was assessed, and disagreements were resolved by a third radiologist.

### Intraoperative findings and pathological evaluation

The degree of CA invasion was assessed intraoperatively. The cervical artery was considered to be invaded if the tumor encased or occluded the CA, and no blood signal was detected by intraoperative ultrasonography. The CA was considered not invaded if it could be separated from the tumor. Intraoperative findings and pathological assessments are considered the gold standard.

### Statistical analysis

The dataset was randomly and equally divided into a training and a validation set. Interobserver agreement for CT features was evaluated by calculating the kappa value, and interobserver agreement was evaluated using the intraclass correlation coefficient (ICC). Using intraoperative findings or pathological diagnosis as the reference, univariate and multivariate logistic regression analyses were performed using the training set to determine the predictive factors of CA invasion. A function based on the variance inflation factor was used to check the collinearity of the variables included in the regression equation, and a variance inflation factor greater than 10 indicated multicollinearity (21). Based on the results of the multivariate analysis, a model for CA involvement was constructed using the training set. The discrimination and calibration of the model were evaluated in the entire dataset.

The diagnostic performance of the model was evaluated by receiver operating characteristic curve (ROC) analysis. Sensitivity, specificity, PPV, and negative predictive value with 95% confidence intervals (CI) were calculated. The areas under the ROC curve (AUCs) were compared using the DeLong method.

The model was built as a nomogram to stratify the individual risk of CA invasion. The agreement between the predicted and actual probability of CA invasion was determined using a calibration curve. Goodness of fit was assessed using the Hosmer-Lemeshow test. Clinical utility was evaluated using decision curve analysis (DCA) by quantifying the net benefits of the model in the validation set. Patients were classified into a high-risk and a low-risk group, and the threshold was identified by ROC analysis. Analyses stratified by clinical risk in the entire dataset were performed to explore the potential association of the model with CA involvement.

Statistical analyses were conducted using SPSS version 26.0 (IBM, Armonk, NY, USA) and R software version 3.4.4 (www.r-project.org). A two-sided p-value of less than 0.05 indicated a significant difference.

## Results

### Patient characteristics and rate of CA invasion

The mean age of our cohort (140 men and 75 women) was 53.5 years. The incidence of CA invasion in the training and validation groups was 27.36% and 22.94%, respectively. Clinical and imaging characteristics in the two groups are listed in **Table 1**. The pathological types of head and neck masses are shown in **Supplementary Table 1** and **2**. The results showed that 98.6% of the tumors were malignant, and 1.4% were benign.

**Table 1:** Characteristics of patients in the training and validation cohorts.

Characteristic	Training cohort	Validation cohort	P value
	(n = 106)	(n = 109)	
Age (y)*	53.99 ± 12.84 (24-82)	53.08 ± 13.58 (12-82)	0.62
**Gender**			
Male	63	77	0.09
Female	43	32	
**Lymph node**			0.01
Yes	70	89	
No	36	20	
**Side of carotid artery**			0.95
Left	52	53	
Right	54	56	
**Perivascular fatty gap**			0.60
No	64	62	
Yes	42	47	
**Vascular displacement**			0.16
No	54	66	
Yes	52	43	
**Vascular deformation**			0.09
No	83	95	
Yes	23	14	
**ASA (°)**	140.84 ± 97.31 (0-360)	122.12 ± 91.36 (25-360)	0.15
**LAA (°)**	54.57 ± 28.81 (0-139)	51.45 ± 26.05 (14-123)	0.41
**LLA (mm)**	26.06 ± 18.84 (2.1-105)	23.59 ± 18.01 (4-119.1)	0.33
**LTLA (mm)**	35.83 ± 16.99 (2.4-90)	35.45 ± 18.55 (11-124.6)	0.88
**LLA/LTLA**	0.70 ± 0.35 (0.17-3.28)	0.64 ± 0.22 (0.17-1.00)	0.17
**Report diagnosis**			0.32
No	64	73	
Yes	42	36	
**Invasion**			0.46
No Yes	77 29	84 25	

*Data are mean ± standard deviation; data in parentheses are range. P > 0.05 suggests no significant difference between the subjects in the two cohorts. ASA axial surrounding angle (degree of encasement of vascular structure), LAA longitudinal axis angle (angle between the mass and the vascular structure longitudinal of the vascular structure), LLA length of interface between tumor and carotid artery along the long axis of vascular structure, LTLA length of tumor along the long axis of vascular structure, LLA/LTLA the ratio of length of interface between tumor and carotid artery along the long axis of vascular structure to length of tumor along the long axis of vascular structure.

### Interobserver agreement for CT signs

Interobserver agreement for perivascular adipose tissue, vascular abutment, and vascular deformation was good to excellent (22) (κ=0.77-0.84), and the interobserver agreement for arterial encasement, the angle between tumors and CA, length of the interface between tumors and CA, and tumor length along the CA was excellent (23) (ICC = 0.91-0.96; 95% CI, 0.62-988).

### CT findings that could predict CA involvement

Imaging features that could predict CA involvement are shown in **Table 2**. On univariate analysis, vascular abutment, vascular deformation, artery encasement, the angle between tumors and CA, tumor length along the CA, and the length of the interface between tumors and the CA divided by tumor length along the CA were significantly more common in the group with CA involvement (p<0.05). On multivariate analysis, vascular deformation (odds ratio [OR], 7.17; 95% CI, 1.53-39.80; p =0.02) and arterial encasement (OR, 1.02; 95% CI, 1.01-1.03; p<0.001) were independently and significantly associated with CA involvement.

**Table 2:** Important imaging findings for prediction of carotid artery involvement.

	Univariate	Analysis		Multivariate	Analysis	
	Coefficient	OR (95%CI)	p	Coefficient	OR (95%CI)	p
Age	-0.35	0.70 (-0.19-1.59)	0.40			
Gender	0.03	1.03 (0.996-1.07)	0.09			
Lymph node	-0.18	0.83 (-0.08-1.75)	0.70			
Side of carotid artery	-0.15	0.86 (0.01-1.72)	0.74			
Perivascular fatty gap	-19.38	3.84e-09 (-3252.39-3252.39)	0.99			
Vascular displacement	1.85	6.34 (5.33-7.35)	<0.001*	0.40	1.48 (0.33-6.75)	0.60
Vascular deformation	2.51	12.31 (11.24-13.38)	<0.001*	1.97	7.17 (1.53-39.80)	0.02*
ASA	0.02	1.02 (1.01-1.03)	<0.001*	0.02	1.02 (1.01-1.03)	<0.001*
LAA	0.04	1.04 (1.02-1.06)	<0.001*	0.003	1.00 (0.97-1.03)	0.84
LLA	0.035	1.04 (1.01-1.11)	0.004*	-0.04	0.96 (0.90-1.02)	0.23
LTLA	0.02	1.02 (0.10-1.05)	0.08			
LLA/LTLA	2.46	11.75 (9.76-13.74)	0.02*	2.26	9.54 (0.37-169.05)	0.11

ASA axial surrounding angle (degree of encasement of vascular structure), CI confidence interval, LAA longitudinal axis angle (angle between the mass and the vascular structure longitudinal of the vascular structure), LLA length of interface between tumor and carotid artery along the long axis of vascular structure, LTLA length of tumor along the long axis of vascular structure, LLA/LTLA the ratio of length of interface between tumor and carotid artery along the long axis of vascular structure to length of tumor along the long axis of vascular structure, OR odds ratio. * indicates significant difference.

The diagnostic performance of the model is shown in **Table 3**. The extent of encasement predicted CA involvement. AUC analysis showed that the threshold for encasement was 178°, such that values larger than 178° suggested CA involvement.

**Table 3:** Diagnostic performances of the CT imaging findings.

CT imaging findings	Training set					Validation set				
	SEN	SPE	ACC	PPV	NPV	SEN	SPE	ACC	PPV	NPV
Vascular deformation	0.55	0.91	0.81 (0.72-0.88)	0.70	0.84	0.32	0.93	0.79 (0.70-0.86)	0.57	0.82
ASA	0.79	0.88	0.86 (0.78-0.92)	0.72	0.92	0.68	0.94	0.88 (0.80-0.93)	0.77	0.91
Model	0.86	0.87	0.87 (0.79-0.93)	0.71	0.94	0.68	0.92	0.86 (0.78-0.92)	0.71	0.91

Prediction model, including vascular deformation and the degree of encasement of carotid artery. ACC, accuracy; ASA, axial surrounding angle (degree of encasement of vascular structure); SEN, Sensitivity; SPE, Specificity; PPV, positive prediction value; NPV, negative prediction value.

### Model development and validation

**Figure 2:**
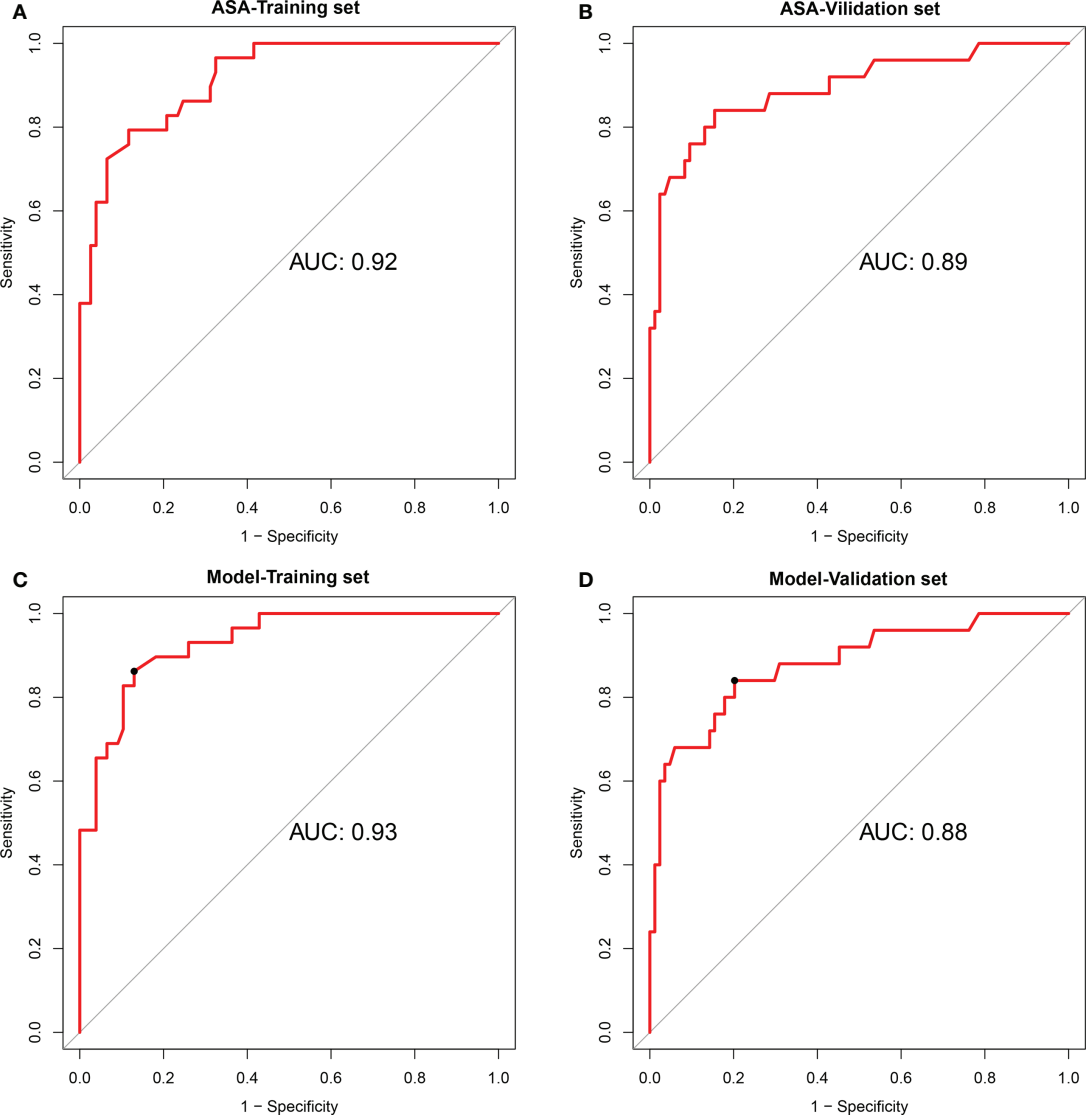
Performances of the degree of encasement of the carotid artery and the prediction model in the training cohort and validation cohort. **(A, B)** degree of encasement of vascular structure (axial surrounding angle), **(C, D)** Prediction model, including vascular deformation and the degree of encasement of the carotid artery. ASA, axial surrounding angle.

**Figure 3:**
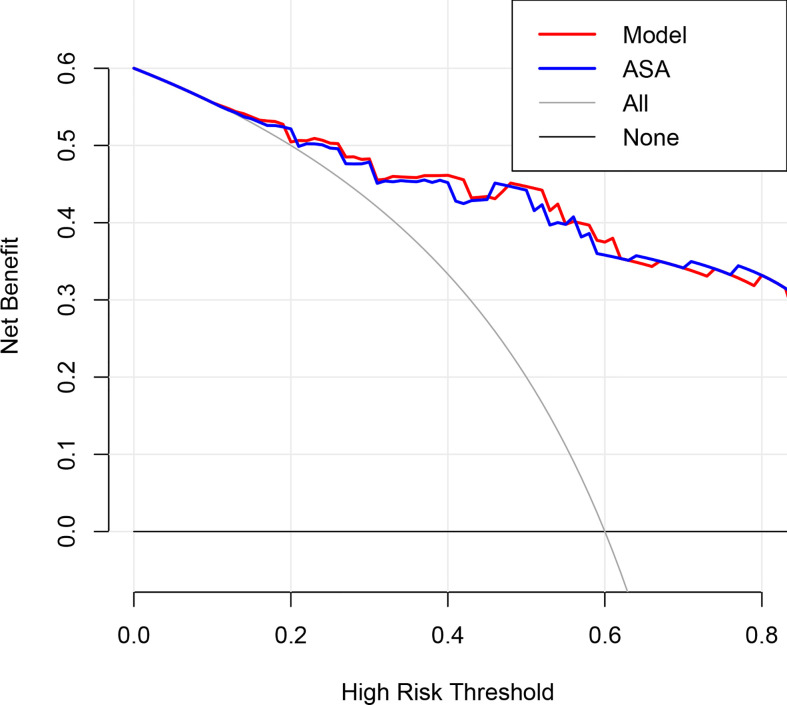
Decision curve analysis for the degree of encasement of the carotid artery and the prediction model in the validation dataset. The y-axis measures the net benefit, which is calculated by summing the benefits (true-positive findings) and subtracting the harms (false-positive findings), weighting the latter by a factor related to the relative harm of undetected carotid artery invasion compared with the harm of unnecessary treatment. ASA, axial surrounding angle.

The AUC of the extent of encasement to predict CA involvement in the training and validation set was 0.92 (95% CI, 0.86-0.97) and 0.89 (95% CI, 0.81-0.97), respectively. A regression coefficient-based model containing two potential risk factors—vascular deformation and encasement—was constructed based on the results of the multivariable logistic regression analysis in the training set (**Supplementary Table 3**). The AUC of this model in the training and validation set was 0.93 (95% CI 0.88-0.98) and 0.88 (95% CI 0.80-0.96), respectively (**Figure 2**). The variance inflation factor of these two factors was 1.284, indicating no multicollinearity. The AUC of the extent of vascular encasement was marginally higher (p=0.07) than that of the prediction model in the validation cohort. DCA demonstrated that the degree of arterial encasement and the model provided were more beneficial across the range of threshold probabilities than treat-all and treat-none strategies in both cohorts, and no obvious difference was observed (**Figure 3**).

**Figure 4:**
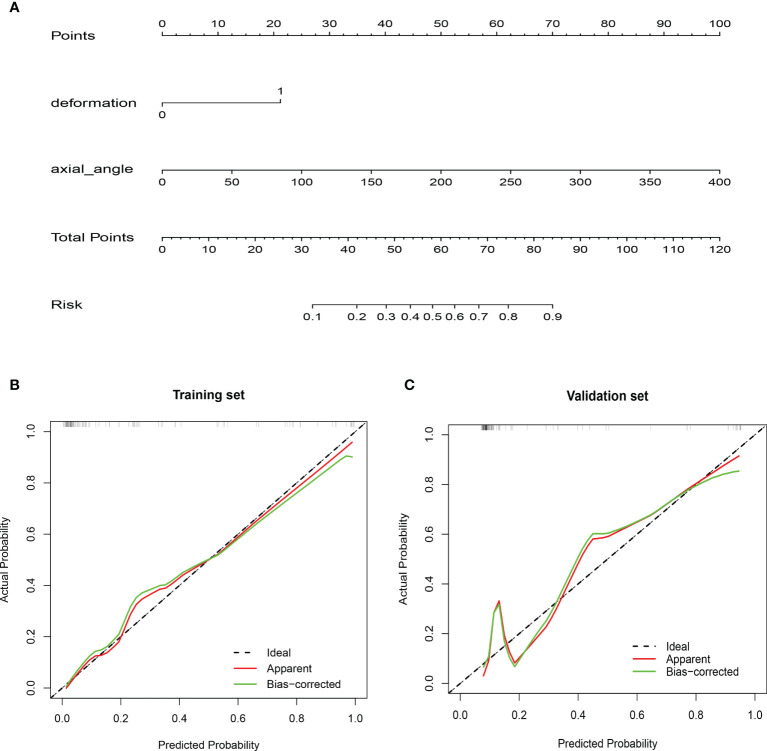
Nomogram and calibration curves. **(A)** A nomogram was constructed in the training cohort, with vascular deformation and the degree of encasement of the carotid artery incorporated. Each risk variable was listed separately on the nomogram with a corresponding number of points assigned to a given variable magnitude. Then, the cumulative point score for all variables matched the “diagnostic possibility,” which was the carotid artery invasion probability of the patient. Calibration curves of the nomogram in the **(B)** training and **(C)** validation cohorts. The calibration curve of the prediction model demonstrated that the estimated risks were in agreement with the observed risks of carotid artery invasion in both the training and validation sets.

A nomogram including vascular deformation and encasement was constructed (**Figure 4A**). Each risk factor was included in the nomogram and scored. The total score for each patient was based on the predicted probability of arterial invasion. The calibration curve of the model demonstrated that the estimated risks agreed with the observed risks of CA involvement in the training and validation sets (**Figures 4B, C**). In addition, there was no significant difference in the results of the Hosmer-Lemeshow test (p=0.72 and 0.38), suggesting adequate goodness of fit.

Using 0.248 as the cutoff score derived from the AUC of the model in the training cohort, patients were classified into a high-risk group (score ≥ 0.248) and a low-risk group (score < 0.248). The patients with a lower risk generally had a lower probability of arterial invasion than those with higher risk scores (**Supplementary Figure 1**). After stratification by clinicopathological risk factors, the model’s performance was excellent in all subgroups (AUC=0.83-1.00) (**Supplementary Figure 2**). Moreover, arterial encasement of 178° accurately predicted CA involvement in these subgroups (**Supplementary Figure 3**).

## Discussion

The results of this study revealed CA deformation and encasement as significant and independent predictors of arterial invasion by head and neck tumors. A model containing these two potential risk factors was developed, and its predictive performance in the validation set was satisfactory (AUC, 0.88; sensitivity, 68%; specificity, 92%). A nomogram was created to calculate the probability of CA invasion. Furthermore, the model was validated internally, and its discrimination and calibration were assessed.

It is crucial to perform preoperative examinations to assess the risk of resecting carotid arteries adjacent to tumors. However, the radiological criteria for diagnosing carotid invasion are conflicting. For instance, Pons et al. (12) found that pathological deformations of the CA were the most significant predictor of vascular wall invasion in all resected cases. In addition, the predictive accuracy of CA deformation or arterial encasement of >180° for arterial invasion was 79.7-92.3% (9, 11, 12, 24). In our model, the predictive accuracy of vascular deformation was slightly lower, possibly because this condition was observed in only 17.2% of our cohort. Furthermore, our results showed that encasement of >178° suggested arterial invasion. Encasement was 178-180° in seven cases (3.3%). Some studies reported that CA involvement of ≥270° predicted arterial wall invasion with a sensitivity of 92-100% and a specificity of 88-93% (8, 25, 26). In 44.4% of our cases, encasement greater than 270° had direct tumor invasion. Despite the lack of consensus on the extent of encasement that can predict arterial wall invasion, 180 degrees is considered the threshold Despite the lack of consensus on the extent of encasement of the internal CA, 180° is considered the threshold. Some authors observed that CA dissection was unfeasible in cases where encasement was greater than 270° (8, 11, 27). However, encasements greater than 178° in the absence of other signs of massive invasion of the CA are not a contraindication for CA dissection.

The AUC, sensitivity, and specificity of the logistic regression model containing two potential risk factors—arterial wall deformation and vascular encasement—to predict CA invasion were 0.88, 0.68, and 0.92 in the validation cohort. These values are similar to those of encasement of >178°, suggesting that combining these features did not improve the prediction of arterial invasion based on preoperative CT imaging findings. Although these results seem counterintuitive, the sensitivity and PPV of arterial wall deformation in predicting arterial invasion were low because deformation was observed in only 23 (42.6%) patients with encasement of >178°, and combining these two factors reduced the predictive power of the extent of encasement.

Our results suggest that the extent of encasement could predict CA invasion in cases in which encasement was greater than 178° and CA deformation was not observed on CT images, whereas a prediction model should be used in other cases. The nomogram based on two preoperative CT findings can predict CA invasion by head and neck tumors. This model showed good discrimination and calibration, and the estimated risks agreed with the actual risks in the training and validation sets. DCA in the validation set showed that, except in a small range of threshold probabilities, interventions based on this model were more beneficial than treat-all and treat-none approaches. Moreover, after stratification by clinical risk factors, the model’s performance was good in all patient subgroups.

Previous studies analyzed CT signs of peripancreatic arterial and venous invasion in pancreatic carcinoma, portal vein invasion in gallbladder cancer, and hepatic arterial invasion by hilar cholangiocarcinoma (13–15, 28, 29). Based on the results of these studies, we hypothesize that the encasement to the CA can predict tumor invasion of the wall of different arteries, despite the lack of consensus on the extent of encasement that can predict invasion.

This study had some limitations. First, the retrospective design was a potential source of bias, and the sample size was small because of the lack of standardized multicenter databases. Second, lymph nodes were the majority of neck masses, and carotid contact from the primary neck masses was the minority. In addition, owing to randomization, there was a statistical difference in the factor of whether the head and neck mass was lymph node in the training and validation datasets (p=0.01). This factor was not an independent predictor of CA invasion but should be considered in future studies. Only a few neck masses were benign tumors (1.4%), precluding the development of a model that could differentiate between benign and malignant neck tumors. Third, the model was not validated externally using an independent test set. The generalizability of the results of this study must be verified. Fourth, the predictive value of MRI and ultrasound should be further assessed, and difficult cases should be evaluated using multiple diagnostic approaches.

In conclusion, preoperative CT findings, including vascular deformation and extent of CA encasement, can help predict CA invasion by head and neck tumors. In addition, the model containing these two potential risk factors exhibited good discrimination and calibration, with potential clinical application.

## Data availability statement

The original contributions presented in the study are included in the article/**Supplementary Material**. Further inquiries can be directed to the corresponding author.

## Ethics statement

The studies involving human participants were reviewed and approved by ethics committee of National Cancer Center/Cancer Hospital, Chinese Academy of Medical Sciences and Peking Union Medical College. Written informed consent from the participants’ legal guardian/next of kin was not required to participate in this study in accordance with the national legislation and the institutional requirements.

## Author contributions

YZ, DB, and DL, conception and design. DL and YZ, administrative support. YZ, DB, XW, ML, LL, ZZ, XZ, and DL, provision of study materials or patients. YZ, DB, and XW, collection and assembly of data. YZ, DB, ML, and LL, data analysis and interpretation. DB, writing-original draft. DB, YZ, ZZ, XZ, and DL, draft editing and visualization. All authors contributed to the article and approved the submitted version.

## Funding

This work was supported by the Non-profit Central Research Institute Fund of Chinese Academy of Medical Sciences (2019XK320073).

## Conflict of interest

The authors declare that the research was conducted in the absence of any commercial or financial relationships that could be construed as a potential conflict of interest.

## Publisher’s note

All claims expressed in this article are solely those of the authors and do not necessarily represent those of their affiliated organizations, or those of the publisher, the editors and the reviewers. Any product that may be evaluated in this article, or claim that may be made by its manufacturer, is not guaranteed or endorsed by the publisher.

## Abbreviations

AUC, Areas under the curve; CA, carotid artery; CI, confidence interval; CT, computed tomography; DCA, Decision curve analysis; ICA, internal carotid artery; ICC, intraclass correlation coefficient; MIP, maximum intensity projection; MPR, Multiple planar reconstruction; MR, magnetic resonance; OR, odds ratio; ROC, receiver operating characteristic curve.
