# Supplementary material for: Prediction model based on preoperative CT findings for carotid artery invasion in patients with head and neck masses
Source: Front Oncol. 2022 Oct 6;12:987031. doi: 10.3389/fonc.2022.987031 (PMC9582344; doi:10.3389/fonc.2022.987031)
Supplement: Supplementary file 1 [file DataSheet_1.pdf]

Supplementary Material

1 Supplementary Figures and Tables

1.1 Supplementary Figures

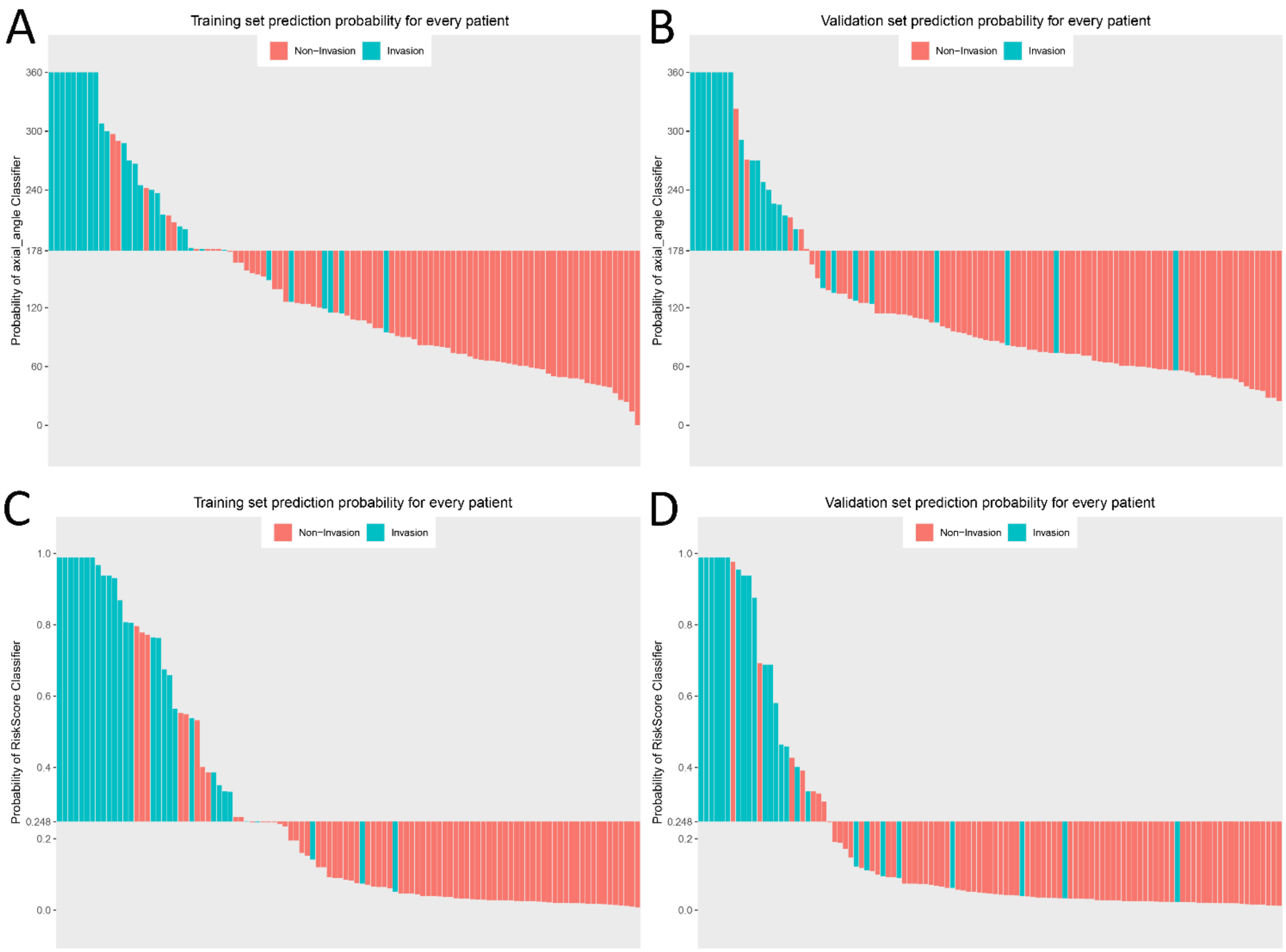

Supplementary Figure 1. Risk score for every patient in the training and validation cohort respectively. (A, B) Based on the degree of encasement of the carotid artery classifier, (C, D) Based on the prediction model-based classifier.

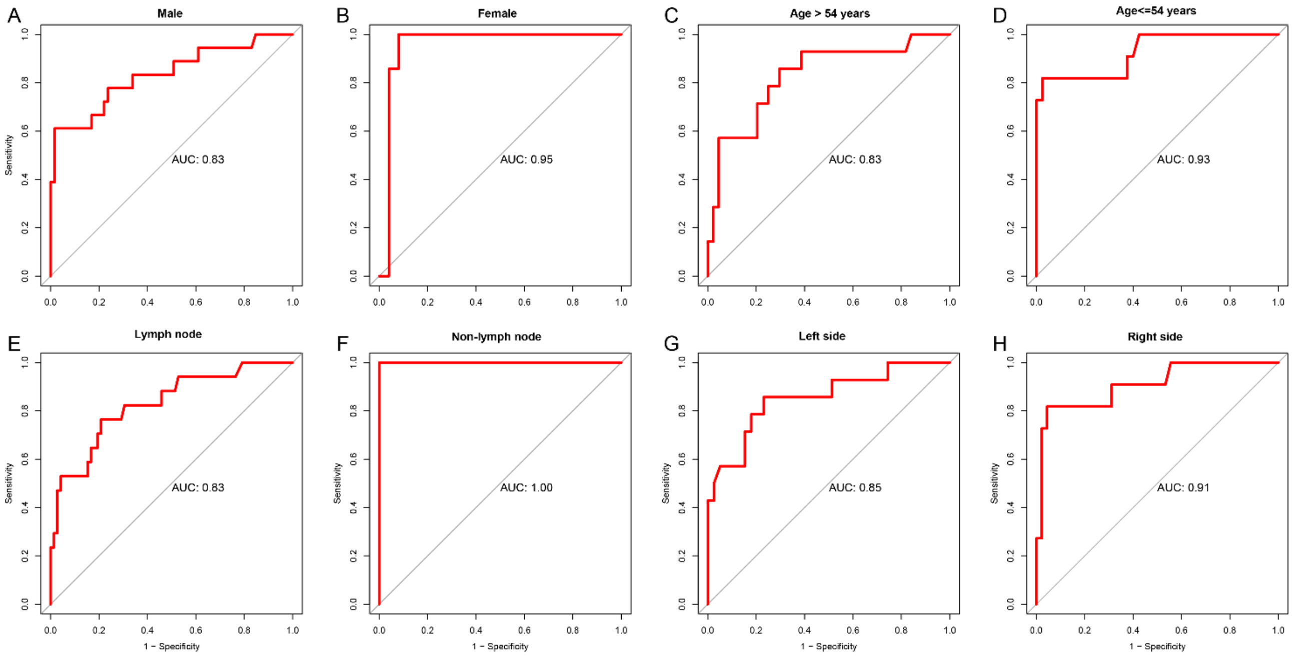

Supplementary Figure 2. The performances of the prediction model within different clinical-pathologic subgroups. ROC analysis with the AUC to evaluate the prediction model as an independent biomarker in the following clinical-pathologic factors respectively: (a, b) gender (male or female), (c, d) age (>54 or ≤54), (e, f) lymph node or non-lymph node, (g, h) left side or right side carotid artery.

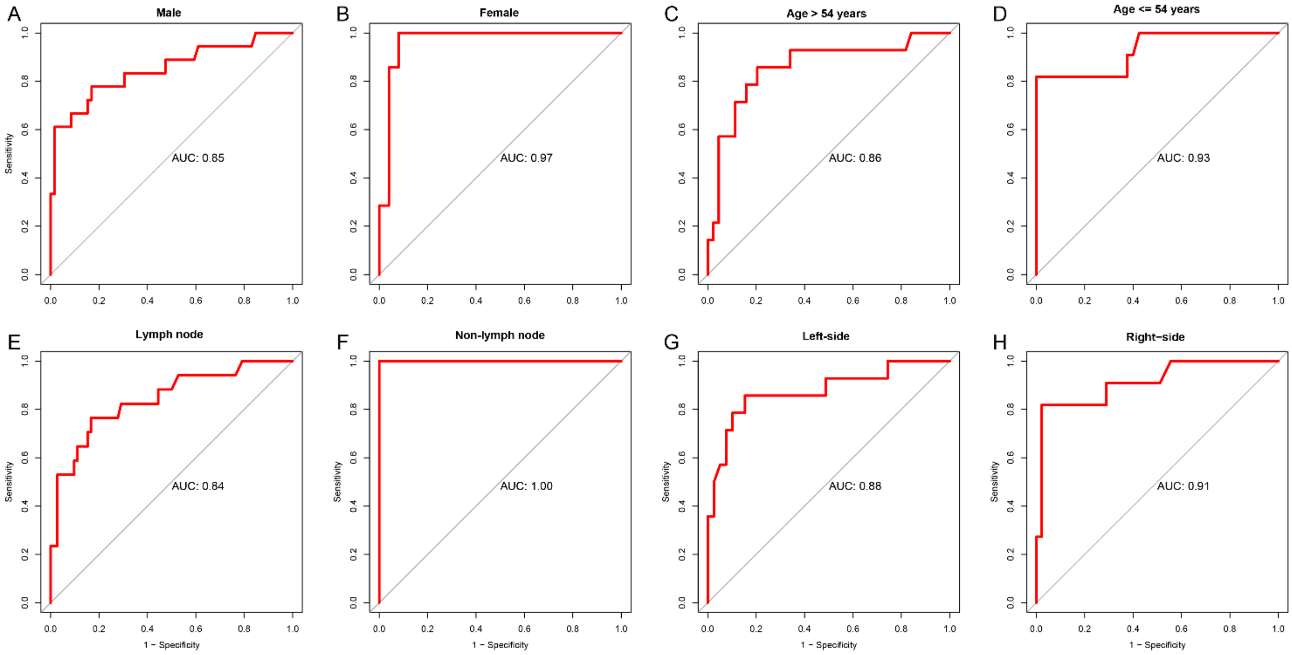

Supplementary Figure 3. The performances of the 178 degrees of circumferential involvement within different clinical-pathologic subgroups. ROC analysis with the AUC to evaluate the prediction model as an independent biomarker in the following clinical-pathologic factors respectively: (a, b) gender (male or female), (c, d) age (>54 or ≤54), (e, f) lymph node or non-lymph node, (g, h) left or right carotid artery.

1.2 Supplementary Tables

Supplementary Table 1. Pathological types of the head and neck masses in the training and validation cohorts

**Pathological types**	**Training Cohort (n=106)**	**Validation Cohort (n=109)**	**p value 0.10**
**Metastatic lymph node**			
Non-cervical or unknown primary	24	14	
Thyroid carcinoma	29	24	
Nasopharyngeal carcinoma	4	2	
Laryngeal carcinoma	5	8	
Hypopharyngeal carcinoma	5	15	
Tongue cancer	3	4	
**Lymphoma**	1	2	
**Carcinoma of mouth floor**	0	1	
**Nasopharyngeal carcinoma**	1	1	
**Oropharyngeal cancer**	3	2	
**Hypopharyngeal carcinoma**	3	6	
**Laryngeal carcinoma**	5	5	
**Thyroid cancer**	18	17	
**Neurofibroma**	0	3	
**Liposarcoma**	0	3	
**Malignant melanoma**	5	2	

Note. — $P > 0.05$ suggests no significant difference between the subjects in the two cohorts.

Supplementary Table 2. Pathological types of the head and neck masses

Pathological types	Benign (n=3)	Malignant (n=212)
**Metastatic lymph node**		
Non-cervical or unknown primary		38 (17.7)
Thyroid carcinoma		53 (24.7)
Nasopharyngeal carcinoma		6 (2.8)
Laryngeal carcinoma		13 (6.0)
Hypopharyngeal carcinoma		20 (9.3)
Tongue cancer		7 (3.3)
**Lymphoma**		3 (1.4)
**Carcinoma of mouth floor**		1 (0.5)
**Nasopharyngeal carcinoma**		2 (0.9)
**Oropharyngeal cancer**		5 (2.3)
**Hypopharyngeal carcinoma**		9 (4.2)
**Laryngeal carcinoma**		10 (4.6)
**Thyroid cancer**		35 (16.3)
**Neurofibroma**	3 (1.4)	
**Liposarcoma**		3 (1.4)
**Malignant melanoma**		7 (3.2)

Note. —Data in parentheses are percentages.

Supplementary Table 3. Multivariable logistic regression predicting likelihood of carotid artery involvement

**Variable**	**β**	**SE**	**Wald**	**P**	**OR**	**95%CI**	
						**lower**	**upper**
**Vascular deformation**	1.80	0.70	6.57	0.01*	6.07	1.56	25.58
**ASA**	0.02	0.005	21.10	<0.001*	1.02	1.01	1.03

Note. — ASA axial surrounding angle (degree of encasement of vascular structure), CI confidence interval, OR odds ratio, SE standard error. * indicates significant difference.
